# Treating Diabetic Macular Oedema (DMO): real world UK clinical outcomes for the 0.19mg Fluocinolone Acetonide intravitreal implant (Iluvien™) at 2 years

**DOI:** 10.1186/s12886-018-0726-1

**Published:** 2018-02-27

**Authors:** William Fusi-Rubiano, Chandoshi Mukherjee, Mark Lane, Marie D. Tsaloumas, Nicholas Glover, Andrej Kidess, Alastair K. Denniston, Helen E. Palmer, Avinash Manna, Rupal Morjaria

**Affiliations:** 1 Ophthalmology Department, Queen Elizabeth Hospital Birmingham, University Hospitals Birmingham NHSFT, Mindelsohn Way, Birmingham, B15 2TH United Kingdom; 2 Sandwell & West Birmingham NHS Trust, Dudley Road, Birmingham, B18 7QH United Kingdom; 3 Academic Unit of Ophthalmology, Institute of Inflammation & Ageing, University of Birmingham, Edgbaston, Birmingham, B15 2TT United Kingdom

**Keywords:** Diabetic Macular Oedema, Iluvien, Diabetic Retinopathy, Fluocinolone Acetonide implant

## Abstract

**Background:**

To compare visual function and structural improvements in pseudophakic eyes with diabetic macular oedema (DMO) treated with the 0.19mg Fluocinolone Acetonide (FAc) intravitreal implant (Iluvien^TM^) in a ‘real world’ setting.

**Methods:**

A single centre retrospective evaluation of patients with DMO unresponsive to conventional treatment treated with the FAc implant according to UK guidelines. Primary efficacy endpoint was best corrected visual acuity (BCVA); secondary endpoints included optical coherence tomography evaluations of the macula (a) central retinal and (b) peak macular thickness collected at annual time points. Primary safety endpoint was new rise in IOP >27mmHg or glaucoma surgery. Patients with <1 year follow-up were excluded.

**Results:**

Twenty-nine eyes were included, with mean(SD) follow up of 792(270) days. Improvement in BCVA and reduction in macular oedema was noted at all timepoints. Mean improvement in BCVA from baseline was 6 ETDRS letters at year 1(n=29), 6.5L at year 2(n=22) and 11L at year 3(n=6). Mean central retinal thickness at baseline was 451 microns, 337 microns at year 1, 342 microns at year 2 and 314 microns at year 3. Two eyes required IOP-lowering drops post implant. Supplementary treatment for persistence or recurrence of DMO was necessary in 18 eyes over the total study period of 3 years with mean time to supplementary treatment being 12 months.

**Conclusions:**

Our evaluation of the 0.19mg FAc implant delivered in a real-world setting, provides additional evidence that it is effective and safe in the treatment of patients with DMO, and can provide sustained benefit for patients with previously refractory disease.

## Background

Worldwide 422 million people have diabetes [1]. A third of these people have diabetic retinopathy (DR) and of these a further third have vision threatening DR including diabetic macular oedema (DMO) [2]. In developed countries, DMO is a leading cause of blindness in the working population [3]. DMO occurs due to impairment of the blood retinal barrier and increased vascular permeability caused by anatomical and biochemical changes including pericyte loss, endothelial cell dysfunction and increased pro-inflammatory changes [4]. Vascular endothelial growth factor has a major role in these mechanisms, however the role of anti-oxidants, inflammatory agents and angiogenesis has also been shown [5–7].

For many years laser treatment was the mainstay treatment for DMO, at times supplemented by short acting corticosteroid injections (peri/intra-ocular triamcinolone). In more recent years the role of laser has been largely replaced by the use of anti-vascular endothelial growth factor (VEGF) agents (notably bevacizumab, ranibizumab and aflibercept) [8]. A significant proportion of patients with DMO are however unresponsive to anti-VEGF agents. Gonzalez et al found that 39.7% patients treated with anti-VEGF had minimal response of <5 letter gain in best corrected visual acuity (BCVA) after 3 months [9]. This ‘minimal response’ at 3 months was associated with worse long-term BCVA (52 weeks and 156 weeks), which may provide a simple method of identifying sub-optimal DMO responders.

Fluocinolone acetonide [FAc] 0.19mg was approved by NICE in 2013 as a treatment option for pseudophakic patients with chronic DMO that are refractory to other therapies, such as laser and anti-VEGF [10]. The main source of evidence for its efficacy was the Fluocinolone Acetonide in Diabetic Macular Edema (FAME) A and B randomized clinical trials which showed clinical effectiveness of 36 months duration [11, 12]. Although ‘real-world’ data is now emerging, it is still largely limited to the first two years after implantation [8]. The aim of this study was to evaluate the longer-term clinical effectiveness and safety of the FAc implant in patients with DMO treated in the context of a single tertiary centre in the UK.

## Methods

This is a single centre retrospective evaluation of the use of the 0.19mg FAc implant (Iluvien^TM^) in patients with DMO unresponsive to conventional treatment. This evaluation was approved by and registered with the relevant NHS trust (University Hospitals Birmingham NHS Foundation Trust). Patients were assessed for treatment with the FAc implant as guided by NICE (UK) Technology Appraisal (TA301) which restricts its use to refractory DMO in pseudophakic patients. Refractory DMO was determined by clinician and assessed as an inadequate response to conventional therapy (laser and/or anti-VEGF) either no reduction in central retinal thickness or minimal reduction from treatment and a persistence in macula oedema of >250um. Post-FAc implantation, patients continued to be seen regularly to evaluate efficacy and safety, and to monitor associated retinopathy and other ocular disease. For the purposes of this evaluation, the inclusion criteria was all patients at our centre who had been treated with the FAc implant for refractory DMO and for whom there was a minimum of one year follow-up. Data was extracted anonymously from the electronic medical record (Medisoft) in March 2017. The primary efficacy endpoint was best corrected visual acuity (BCVA). Absolute BCVA was evaluated in LogMAR but for presentation of change in BCVA this was converted to number of letters to enable direct comparison to the FAME study [13]. Secondary efficacy endpoints included spectral domain optical coherence tomography (SD-OCT) evaluation of the central retinal thickness and peak macular thickness as per the Heyex^TM^ software from Heidelberg Engineering (Heidelberg, Germany), proportion of cases requiring ‘top-up’ treatment, and time from baseline for ‘top-up’ treatment.

The primary safety endpoint was new rise in IOP over 27mmHg or glaucoma surgery. Evaluation was carried out at annual time-points up to 3 years. Patients with less than 1 year follow-up or who had received treatment for other pathology (e.g. for uveitis) were excluded.

### Statistical Analysis

BCVA, mean central retinal thickness and mean peak macular thickness were evaluated against baseline for each time-point using student’s unpaired t test for parametric data and Mann-Whitney test for non-parametric data. P-values were calculated with a value of less than 0.05 taken to indicate statistical significance. Statistical analysis was performed using SPSS version 20.

## Results

### Demographics

Overall 37 eyes (33 patients) were treated with the FAc implant between January 2014 and March 2016. Four eyes (4 patients) were excluded due to being treated for non-DMO diagnosis and a further 4 eyes (2 patients) were excluded due to having less than 1 year follow-up.

Of the 29 eyes (27 patients) included, mean age of the patients was 69.1 (range, 44-90) with an equal distribution of gender (13 females, 14 males) and laterality of eye treated (Right eye 17, left eye 12). Mean baseline BCVA was 0.77 and 97% were pseudophakic (28 of 29 eyes). Three eyes (2 patients) received the FAc implant after laser treatment alone due to unsuitability for use of anti-VEGF post recent stroke/heart disease. Three patients (3 eyes) had had previous vitrectomy. Twenty of 29 eyes had a duration of DMO more than 3 years prior to implant. Full data outcomes were available for 29 eyes at year 1, 20 at year 2 and 6 at year 3. Mean (SD) duration of DMO prior to treatment was 2.6 (0.77) years; in the subset for which 3 year data is available the duration of DMO was more than 3 years in all cases with mean (SD) duration 3.2 (0.31) years.

All patients had received either laser and/or anti-VEGF prior to treatment with the FAc implant (Table 1). Twenty six eyes had at least 1 prior laser therapy, 17 eyes had at least 1 prior ranibizumab injection, 19 eyes had at least 1 prior bevacizumab injection and 6 eyes at least 1 prior treatment with triamcinolone. 10 eyes had treatment with both ranibizumab and bevacizumab. All 3 eyes unsuitable for anti-VEGF had prior treatment with triamcinolone injection. Minimum time to FAc implant from prior treatment was 8 weeks.

**Table 1:** Prior therapies, number treated and mean number of treatments for all eyes

Prior therapy	Number of eyes treated	Mean number of treatments	Range
Focal/grid macula laser	10	1.15	1-3
Ranibizumab	17	3.94	1-8
Bevacizumab	18	4.32	1-13
Triamcinolone	16	2.31	1-6

### Efficacy endpoints: Best corrected visual acuity

**Fig. 1:**
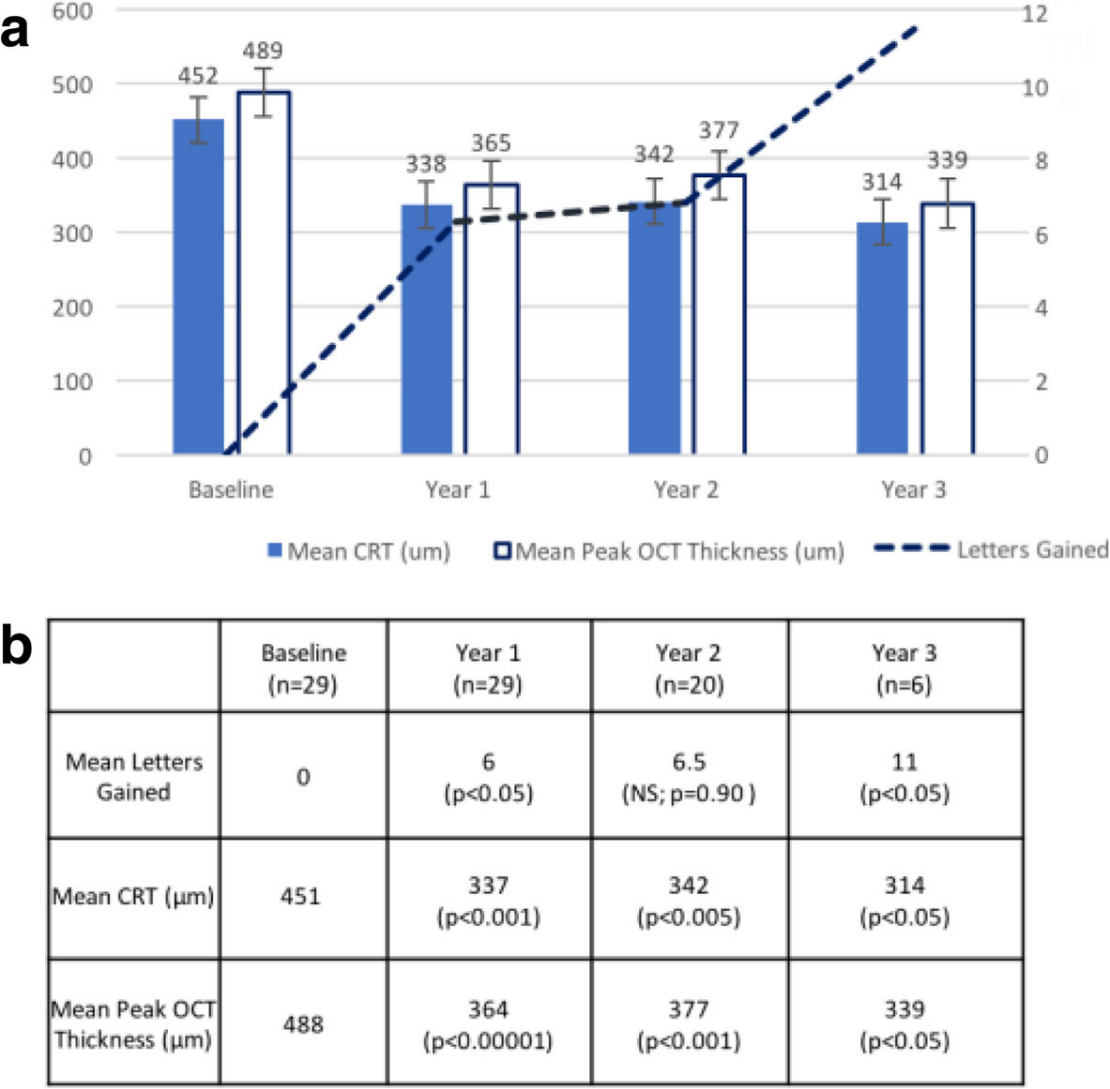
Improvement in BCVA and reduction in retinal thickness at 1, 2 and 3 years after treatment with FAc implant. Graph (**a**) showing mean values for ETDRS letters gained, mean (SD) central retinal thickness (CRT) and peak macular thickness as measured by SD-OCT. Significance at each time-point was tested against baseline (**b**)

Mean (SD) BCVA at baseline was 0.77 (0.37) for all 29 eyes. BCVA improved at all time points with mean (SD) letter gain of 6 (15) at 1 year (p<0.05), 6.5 (15) at 2 years (p=0.90) and 11 (7) at 3 years (p<0.05) after implantation (Fig. 1). Of the 6 patients with at least 3 years follow-up, three eyes (3 patients) had an improvement of 15 letters or more at 3 years from baseline.

### Efficacy endpoints: Retinal thickness

**Fig. 2:**
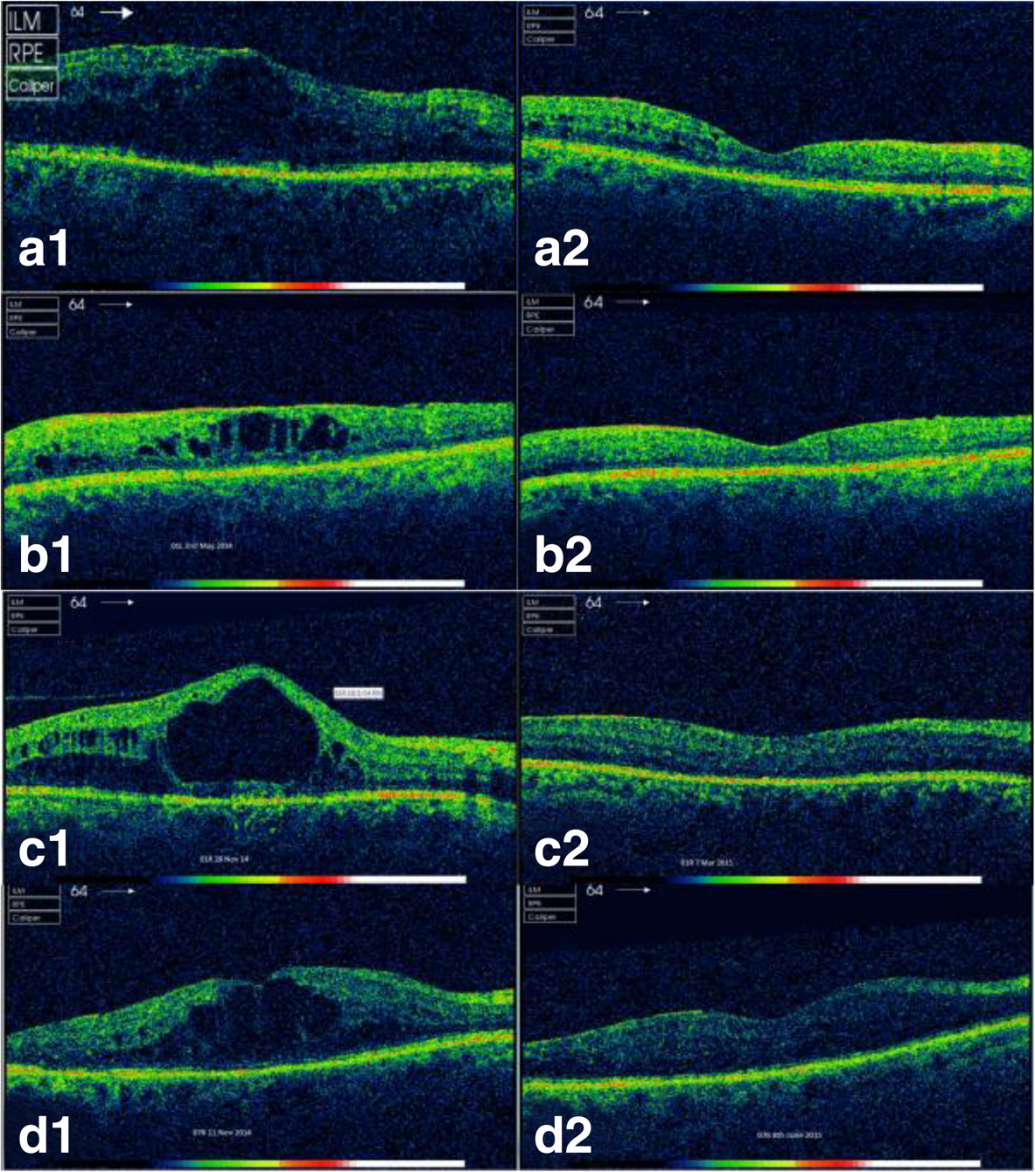
SD-OCT images pre- and post-FAc implant in patients with chronic DMO. **a**. Right eye of 42 year old male with type 1 diabetes and a 4 year history of DMO treated with previous anti-VEGF, triamcinolone and grid laser. DMO (***a***1) resolved by 8 months (***a***2) **b**. Left vitrectomised eye of a patient with 5 year history of DMO. Treated with anti-VEGF, triamcinolone and grid laser. DMO (***b***1) resolved by 5 months (***b***2). **c**. Right eye of 45 year old male chronic non attender with type 1 diabetes and a 1.2 year history of DMO treated with anti-VEGF. DMO (***c***1) resolved by 3 months (***c***2). **d**. Left vitrectomised eye of 53 year old female with type 1 diabetes and 1 year history of DMO treated with anti-VEGF. DMO (***d***1) resolved by 5 months (***d***2)

Mean central retinal thickness at baseline was 451 microns, and mean peak retinal thickness was 488 microns. There was a reduction in both central and peak retinal thickness at all time-points relative to baseline (Fig. 1). The mean (SD) reduction in central retinal and peak thickness was 114 (177) (p<0.001) and 124 (160) (p<0.00001) micrometers respectively at year 1, 103 (207) (p<0.005) and 104 (114) (p<0.001) micrometers at year 2, and 65 (162) (p<0.05) and 99 (90) (p<0.05) micrometers at year 3. Of the patients with at least 3 years follow-up, 50% of eyes (50% of patients)  were clinically dry at 3 years from baseline (p<0.05). Case examples demonstrating OCT appearances pre- and post-FAc implant are provided in Fig. 2.

Of the 33 eyes with a clinical diagnosis of DMO treated with the FAc implant (ie including the four eyes excluded from the primary follow-up on the basis of less than one year’s follow-up), 9 eyes (27%) showed no significant improvement in macular oedema with no reduction or minimal reduction in peak or central thickness at any timepoint.

### Safety endpoints

There were 2 eyes (2 patients) with raised intraocular pressure (IOP) >27mmHg post injection one found at 1 month follow up and one at 6 months follow up. Both were controlled with drops alone. These cases had established raised intraocular pressure (IOP) prior to treatment with the FAc implant: one had a previous diagnosis of ocular hypertension (OHT) and one had a previous diagnosis of primary open angle glaucoma (POAG) for which they were under glaucoma specialist follow up and had been controlled with drops alone. No other ocular or systemic side effects were identified for any patient.

### Persistent or recurrence of DMO requiring supplementary treatment

Supplementary treatment for either persistence of DMO (treatment failure) or recurrence of DMO (premature loss of effect) was necessary in 18 eyes. Supplementary treatment was with one or more of laser (n=4), intravitreal triamcinolone (n=3) or anti-VEGF agent (aflibercept n=11; bevacizumab n=4; ranibizumab n=3). No patients had retreatment with the FAc implant. 10 of 29 (34.5%) eyes had required supplementary treatment by 1 year, 12 of 20 (60%) eyes by 2 years and 5 of 6 (83.3%) eyes by 3 years. Mean number of extra treatments needed per eye/year was 2 at year 1, 1.85 at year 2 and 1.66 at year 3. Mean time until supplementary treatment was 12 months (range 2-22 months), with a mean of 2.6 retreatments (range 1 to 9) during the follow-up period. For the subset with 3 year follow-up, 5 out of 6 eyes needed supplementary treatment with anti-VEGF or laser. Mean time to supplementary treatment was 12.8 months in this cohort (range 10-16 months) with mean number of retreatments needed from this point being 5. In the supplementary treatment group (n=18), mean BCVA at baseline was 0.71 (64.5 L) with a mean change in BCVA was -0.18 (9 L gain (p=0.026)) at 1 year, -0.09 (4.5 L gain (p=0.26)) at 2 years, and -0.22 (11 L gain (p=0.047)) at 3 years.

**Fig. 3:**
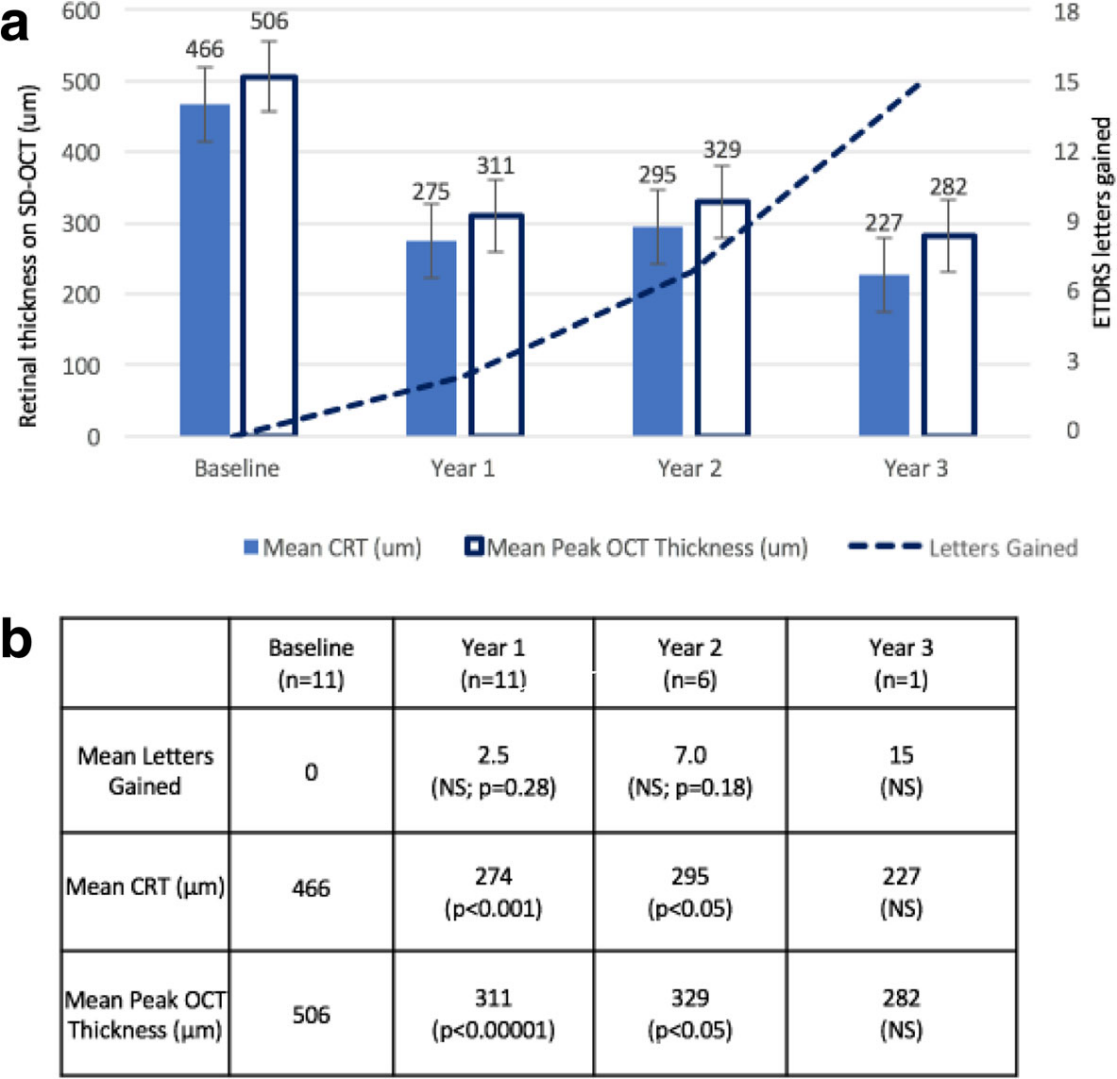
(Supplementary) Improvement in BCVA and reduction in retinal thickness at 1, 2 and 3 years after treatment with FAc implant in those patients who did not require supplementary treatment. Graph (**a**) showing mean values for ETDRS letters gained, mean (SD) central retinal thickness (CRT) and peak macular thickness as measured by SD-OCT. Significance at each time-point was tested against baseline (**b**)

In the group who did not require supplementary treatment (n=11) during the follow-up period mean BCVA at baseline was 0.82 (59 L), with a mean change in BCVA of -0.05 (2.5 L gain (p=0.28)) at 1 year, -0.14 (7 L gain (p=0.18)) at 2 years, and -0.3 (15 L gain) at 3 years (Fig. 3).

### FAc implant in vitrectomised eyes

Three eyes had had previous vitrectomy prior to FAc implant, with one year data being available for 3 eyes, and two year data for 1 eye. Mean (SD) BCVA at baseline was 0.83 (0.06), with a mean change of -0.13 (0.11) (6.5 letter gain (p=0.18)) at 1 year, and -0.13 (0.06) (6.5 letter gain (p=0.06)) at 2 years. Mean (SD) CRT was 326 (70) micrometers at baseline, with a mean change of -55.7 micrometers (116) (p=0.31) at 1 year, and -87.7 (62) (p=0.14) at 2 years. Mean peak macula thickness was 412 (77.2) micrometers thickness at baseline, with a mean change of -63.7 micrometers (81) (p=0.31) at 1 year and -84 (62) (p=0.14) at 2 years. Of the three eyes in this group, one required supplementary treatment at 22 months.

## Discussion

Our study provides some of the first ‘real world’ data through to 3 years follow-up for the effect and safety of the 0.19 mg FAc implant in patients with DMO. Three year data for the FAc implant has hitherto been based almost exclusively on the pivotal FAME study, which demonstrated a 15 letter gain or more at 36 months in over a quarter of patients treated with low dose FAc implant and a reduction of 100 micrometers or more in CRT [11]. The effect on visual acuity was noted to be more significant in those with chronic DMO for more than 3 years compared to the cohort with a more recent diagnosis [11]. Our findings are in line with results of the FAME study, with 50% eyes in our series gaining 15 letters or more and being ‘dry’ on OCT analysis at three years (3 of 6 eligible eyes with three year follow-up data). All three of these eyes had had a duration of DMO more than 3 years. Worse outcomes would be expected in our cohort compared to patients being treated today as patients may have FAc offered at an earlier stage in DMO, whereas many patients in our cohort had a duration of DMO of at least 3 years prior to treatment with FAc implant. A longer duration of DMO and associated disruption to the retinal architecture is known to affect visual outcomes [14].

Other ‘real world’ data of the use of the FAc implant is now emerging. El-Ghrably et al have shown the additional value of treatment with the FAc implant, in patients initially treated with anti-VEGF as BCVA and CRT improved and was maintained at 12 months [15]. In our study the effects were maintained at 36 months in those who responded to the FAc implant. We further evaluated the need for supplementary treatment over the 3-year period which has not yet been reported in real-world studies. Thirty four percent of eyes had required supplementary treatment by 1 year, 60% by 2 years and 83.3% of eyes by 3 years however overall treatments needed was less than or equal to 2 at each year. None of our patients needed retreatment with the FAc implant at 3 years. This significantly lowers the retreatment burden when compared to a recent, large, comparative study of aflibercept, bevacizumab, or ranibizumab which reported that a mean of 9-10 injections were required to control DMO over 12 months [16]. Reduction of injection burden is an important benefit of the FAc implant, as high frequency of intravitreal injections has been shown to affect quality of life and to increase anxiety and work absences in patients with DMO [17]. Most patients want fewer injections and appointments, to achieve the same visual results [17]. Fewer supplementary treatments not only improves the quality of life of these patients, but also contributes to the cost efficacy of the FAc implant.

One question regarding the FAc implant is whether vitrectomised eyes may respond differently. In line with the study by Meireles et al [18] , we found the FAc implant to be effective in vitrectomised eyes. Of the three eyes in our series that had had previous vitrectomies, only one eye needed further treatment during the follow-up, and this was at 1.8 years, compared to the mean supplementary treatment time of 1 year.

The major concerns with the FAc implant are cataract and glaucoma. Cataract occurred in 82-89% of phakic patients by 3 years after implantation of the FAc implant [11] which has led to the NICE (UK) guidelines which restrict its usage to pseudophakic patients with DMO. In our study 97% of eyes were pseudophakic as per the NICE recommendations. Modern cataract surgery is however extremely successful and safe, and thus it may be argued that phakic status should not be a complete bar to treatment if the FAc implant was shown to be otherwise safe and effective. A recent cost analysis has shown that single treatment with the FAc implant is more cost effective than multiple injections of ranibizumab even after allowing for the additional cost of cataract surgery [19]. Although less common than cataract, the greater concern is elevated intraocular pressure (IOP). In FAME, three year data noted an adverse event of elevated IOP of 37% in the standard FAc group (vs 12% in the sham group) and incisional glaucoma surgery being required in 4.8% (vs 0.5% of the sham group). It is interesting to note that our reported adverse events were significantly lower than reported in FAME, although this may in part be due to the relatively smaller number of eyes achieving the three year time-point. In our study only 2 out of 33 eyes were reported to have raised IOP and both of these had a prior history of raised IOP. These patients were successfully treated with drops and did not need surgery. Although Alfaqwi et al have previously reported that there is no additional risk with the FAc implant in patients with well controlled OHT at 12 months [20], further studies are required to evaluate the effect on IOP long-term in patients with OHT and/or POAG.

The primary limitation of our study is its retrospective design and limited numbers, although all data was collected prospectively and recorded on our electronic medical record and imaging database. Use of an electronic medical record platform is also a limitation as limited data is routinely recorded via this platform and therefore limits analysis of crucial factors such as HbA1c and type of diabetes. Use of EMR is however widespread now and is an important method of continuous medical record and source for clinical information in various studies. An additional limitation is that there is variable follow-up, with a diminishing number of patients across the later time-points reflecting the ongoing recruitment to treatment with the FAc implant; this seemed preferable to either limiting the analysis to only that subset which had achieved three years follow-up, or to prematurely censor the follow-up period. Finally we recognize that this is a relatively small study, reflecting its single site nature. The results of the study are however in line with the FAME trials and does provide ‘real world’ support to those results.

## Conclusions

In summary our study is the first to report ‘real world’ clinical outcomes of the therapeutic effects and risk profile of the FAc implant in pseudophakic patients with chronic DMO through to 36 months in UK. There was an improvement in mean VA at all time-points with a mean overall improvement in vision of 8.5 letters at 3 years (p<0.05), associated with a mean reduction in CRT and peak macula thickness. Although three year data was only available for a subset of our patients, 50% of these patients had a 15 letter or more improvement at this time-point, broadly comparable to the 34% with a similar benefit in the FAME study [10]. It should be noted however that almost two-thirds of the eyes in our series required further treatment within three years. The FAc implant appears to provide clinical benefit in pseudophakic patients with chronic DMO that are insufficiently responsive to first line therapies, with a significant proportion of patients benefitting for up to 3 years as shown in the FAME trials [11, 12]. The FAc implant has the added benefit of less frequent visits and fewer injections. It should be considered in all pseudophakic patients with refractive DMO, or considered after laser alone in patients where anti-VEGF is contraindicated. Our outcomes support the findings of the FAME trial that the FAc implant can be safely used in such patients and significantly improve BCVA and reduce macula oedema whilst reducing the overall cost and burden of treatment in this sight-threatening disease.

## Abbreviations

BCVA: Best Corrected Visual Acuity
CRT: Central Retinal Thickness
DMO: Diabetic Macular Oedema
DR: Diabetic Retinopathy
FAc: Fluocinolone Acetonide
IOP: Intraocular Pressure
OHT: Ocular Hypertension
POAG: Primary Open Angle Glaucoma
SD: Standard Deviation
VEGF: Vascular Endothelial Growth Factor
